# Multiplexed gene expression analysis in dissociated and intact planarian flatworm tissues using *in situ* hybridization chain reaction

**DOI:** 10.1242/bio.062669

**Published:** 2026-09-10

**Authors:** Fabienne Dreier, Peter Ditte, Philipp Weiss, Tobias Boothe, Jochen C. Rink

**Affiliations:** Department of Tissue Dynamics and Regeneration, Max-Planck-Institute for Multidisciplinary Sciences, Am Fassberg 11, 37077 Göttingen, Germany

**Keywords:** HCR, *In situ* hybridization, Microscopy, Planarian, *Schmidtea mediterranea*, *Camerata robusta*

## Abstract

Planarians are an important model system for studying whole-body regeneration. The visualization of gene expression patterns during this process is an essential experimental technique to understand the underlying mechanisms. Multicolor fluorescence *in situ* hybridization (FISH) is currently the standard approach for gene expression analysis. However, the small number of available haptens limits probe multiplexing options, and the required sequential development of individual probes can be extremely time consuming. *In situ* hybridization chain reaction (HCR) is a newer technique that promises parallel hybridization of multiple genes via annealing of fluorescently labeled hairpins to target sequences. While HCR is well established in several model organisms, including mice and *Drosophila*, existing protocols perform poorly on whole-mount planarians. Here, we report a systematically optimized HCR protocol that is faster and less labor intensive than traditional FISH. This method can be applied to dissociated planarian cells or combined with the established DEEP-Clear method to label whole-mount animals. These improvements establish HCR as a useful addition to the planarian tool kit, particularly for multiplex gene expression analysis.

## Teaser

**Summary:** We developed a protocol for multicolor *in situ* hybridization chain reaction in dissociated and intact planarian tissue. Coupled with optimized clearing and mounting, our method allows sensitive detection of gene expression in whole-mount specimens.

## INTRODUCTION

Planarians are triploblastic metazoans that occur worldwide in freshwater, terrestrial and marine habitats (Sluys and Riutort, 2018; Vila-Farré and Rink, 2018). Their astonishing ability to regenerate complete animals even from tiny tissue pieces has fascinated scientists for more than 200 years, as illustrated by Dalyell's (1814) observation that planarians can be considered “almost immortal under the edge of the knife”. *Schmidtea mediterranea* is one of the species that display robust whole-body regeneration within only ∼2 weeks and has become the major model species in the field (Drees and Rink, 2023; Newmark and Alvarado, 2001; Reddien, 2018; Saló, 2006; Saló and Baguñà, 2002).

Since transgenesis protocols have not yet been established for planarians, and comparatively few antibodies are available for immunostaining, *in situ* hybridization techniques remain the methods of choice for visualizing the spatiotemporal dynamics of gene expression during tissue homeostasis and regeneration (Agata et al., 1998; Bueno et al., 1997; Forsthoefel et al., 2018; Gaetano and King, 2023; Guerrero-Hernández et al., 2024; King and Newmark, 2013; Oderberg et al., 2017; Pearson et al., 2009; Robb and Sánchez Alvarado, 2002; Umesono et al., 2013). To date, the main techniques have been either chromogenic *in situ* hybridization protocols for single genes or fluorescence *in situ* hybridization (FISH) protocols with peroxidase-catalyzed tyramide amplification for simultaneous visualization of up to three different probes (Bueno et al., 1997; King and Newmark, 2013; Pearson et al., 2009). The drawbacks of tyramide-based FISH are the low number of available haptens and the necessity of developing separate probes sequentially. This adds about one additional day per round of development (King and Newmark, 2013; Pearson et al., 2009). In 2014, an alternative *in situ* hybridization method was developed to overcome these shortcomings: *in situ* hybridization chain reaction (HCR). Instead of the enzyme-catalyzed deposition of fluorescent tyramide molecules, HCR exploits the polymerization of fluorescently labeled and sequence-compatible DNA-hairpin oligos for signal amplification. Specificity is achieved via short antisense probes complementary to the target mRNA that carry hairpin-compatible sequence extensions (Choi et al., 2014, 2018). While the early versions of the probes consisted of a single sequence that served as an initiator for polymerization, newer HCR variations with greater specificity use a pair of probes that must bind next to each other to form the entire initiator sequence (Choi et al., 2014, 2018). Only then can polymerization of a chain of fluorescently labeled hairpins occur, with the length of the chain determining the degree of signal amplification. Additional amplification can be achieved via multiple detection probes for a given target mRNA, and the availability of a range of initiator/hairpin combinations and fluorophore labels allows simultaneous detection of multiple targets in a single polymerization reaction (Choi et al., 2018). Despite these advantages and many successful applications in a range of model species (Choi et al., 2018; Gąsiorowski et al., 2025; Gavriouchkina et al., 2025; Shainer et al., 2023; Watanabe et al., 2024), very few planarian studies have used HCR to date (Dai et al., 2025; Guo et al., 2022; McKinney et al., 2026; Saad et al., 2025), underscoring the need for further technical optimization. Indeed, the generic HCR protocol used with tissues like mouse or chicken embryos (Choi et al., 2018) results in very low signal intensities in planarians in our hands.

Here, we demonstrate major improvements to the existing HCR protocol, resulting in robust and sensitive staining in whole-mount planarians and individualized cell preparations*.* We show that optimization of the permeabilization method and mounting medium, together with washing at lower temperatures, increases the signal-to-noise ratio, thereby establishing HCR as a convenient tool for multiplexed gene expression analysis during regeneration and other biological contexts.

## RESULTS AND DISCUSSION

### HCR on dissociated planarian cell preparations

**Fig. 1.**
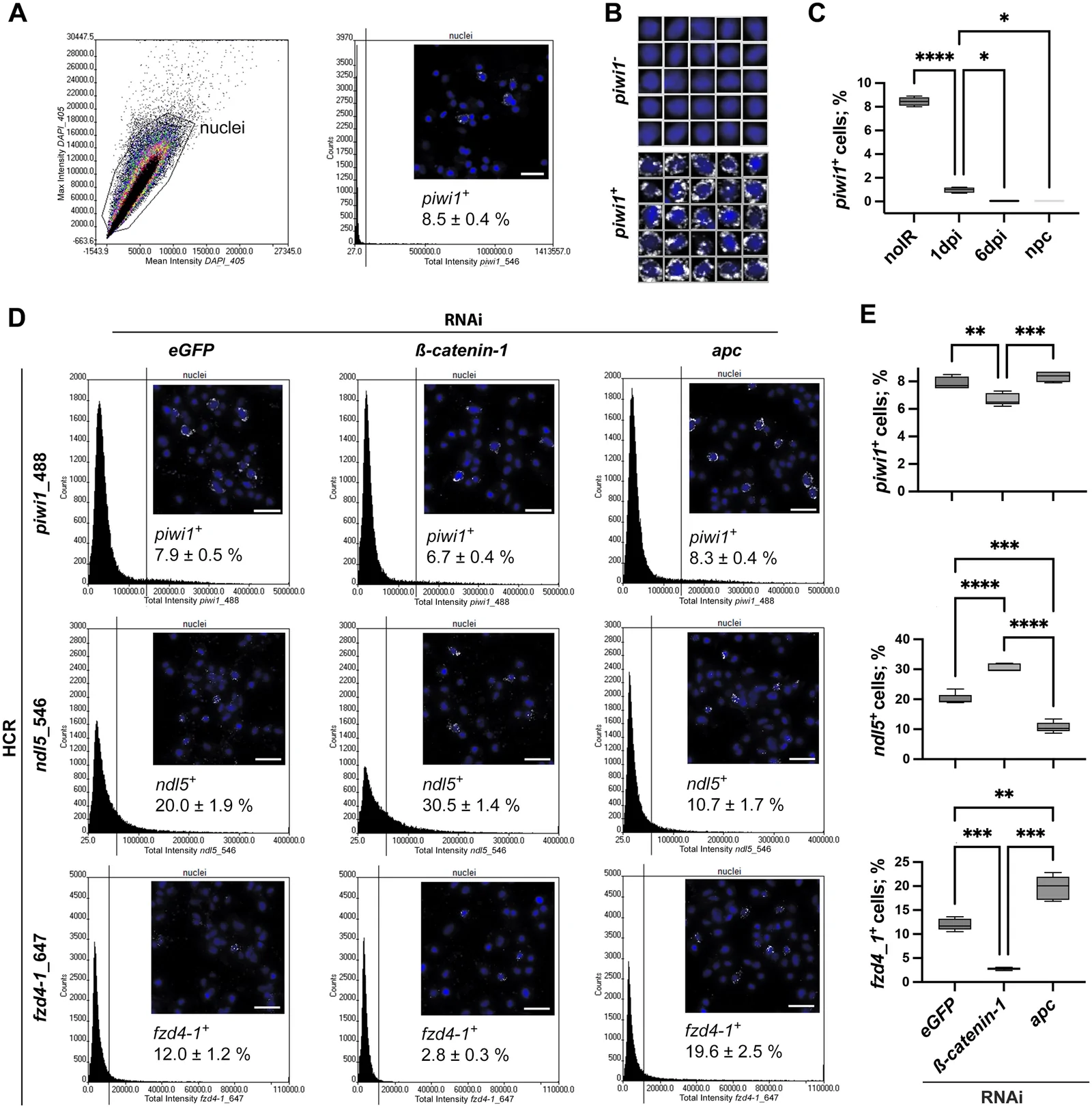
**HCR on dissociated planarian cells.** (A) 8.5% of isolated cells gated by DAPI staining (nuclei) were *piwi1^+^*. Inset shows representative labeling (*piwi1* signal in gray, DAPI in blue). Scale bar: 20 µm. (B) Typical cells from the *piwi1^−^* and *piwi1^+^* gates. (C) Quantification of *piwi1^+^* cells from irradiated worms. Data are from four biological replicates with ten animals each; dpi: days post-irradiation. Non-irradiated worms were used as a control (no IR). (D) Histograms and representative images of HCR-labeled cells from *eGFP*-RNAi (negative control), *apc*-RNAi (posteriorized), and *ß-catenin-1*-RNAi (anteriorized) animals using probes for neoblasts (*piwi1*_488), head/pharynx (*ndl5*_546), and tail (*fzd4-1*_640). Gates were set according to respective negative controls (Fig. S1A). HCR signal in gray, DAPI in blue. Histograms and insets for *eGFP*-RNAi are the same as in Fig. S1A (all RNAi conditions in both figures were tested in the same experiment). Scale bars: 20 µm. (E) Quantification of cells positive for *piwi1*, *ndl5*, and *fzd4-1*. Data are from five biological replicates with ten animals per replicate for each RNAi condition. Boxes represent the values between the 25th percentile and the 75th percentile. Whiskers represent minimal and maximal values. *P<0.05, **P<0.01, ***P<0.001, ****P<0.0001, ANOVA.

The planarian anatomy presents several permeability barriers. Before optimizing a protocol for whole-mount specimens, we therefore began testing the generic HCR v3.0 protocol (Choi et al., 2018) on dissociated planarian cells, easily obtained by macerating animals in acetic-methanol (ACME) solution (García-Castro et al., 2021) and amenable to high-throughput quantitative imaging using multi-well plate assays (Grohme et al., 2023). First, we analyzed specificity by probing *piwi1* (dd_v6_659), a widely used marker of planarian adult stem cells (neoblasts) (Reddien et al., 2005). Importantly, neoblasts can be efficiently depleted in live animals by X-ray irradiation (Baguñà et al., 1989; Reddien et al., 2005). Cells from irradiated worms should therefore be devoid of *piwi1* labeling. After identifying single cells based on their nuclear DAPI intensities (Fig. 1A), we set the gate for the *piwi1*^+^ population according to a negative ‘no probe control’ (npc) (Fig. S1). Using these parameters, around 8% of the cells were *piwi1^+^* in unirradiated animals. This dropped to 1% *piwi1^+^* cells at 1 day after 60 Gy of irradiation (dpi) and 0% *piwi1^+^* cells at 6 dpi, consistent with previous observations (Eisenhoffer et al., 2008) (Fig. 1C). We conclude HCR yields specific labeling in dissociated planarian cells.

To further test HCR on dissociated cells, we used probe sets for the head and pharynx marker *ndl5* (dd_v6_5102) (Scimone et al., 2016) and the tail marker *fzd4-1* (dd_v6_11650) (Stückemann et al., 2017) (Fig. 1D,E). Despite lower mRNA abundance for both genes compared to *piwi1*, we still observed a bright, punctate labeling pattern within a specific subset of cells. RNA interference (RNAi) knockdown of *β-catenin-1* or *apc* resulted in loss of tail or head marker expression, respectively (Stückemann et al., 2017), further supporting the specificity of our HCR approach. The relative size of the *piwi1*^+^ population remained similar in these RNAi conditions, but *ndl5^+^* cells increased in *β-catenin-1*-RNAi compared to the *eGFP*-RNAi control (Fig. 1E), consistent with the formation of ectopic heads (Gurley et al., 2008). Conversely, the number of *ndl5^+^* cells was reduced in *apc*-RNAi animals, which are characterized by ectopic tail formation. Posteriorization was further evidenced by an increase in cells positive for the posterior marker *fzd4-1* (Fig. 1E) (Stückemann et al., 2017).

Dissociated planarian cell labeling has traditionally relied on FISH and immunostaining (Grohme et al., 2023). Together with another recent study (McKinney et al., 2026), our results now establish the general HCR v3.0 protocol as a rapid, specific and sensitive alternative, particularly for multicolor labeling and quantitative expression analysis.

### Optimization of whole-mount fixation and permeabilization steps for HCR

**Fig. 2.**
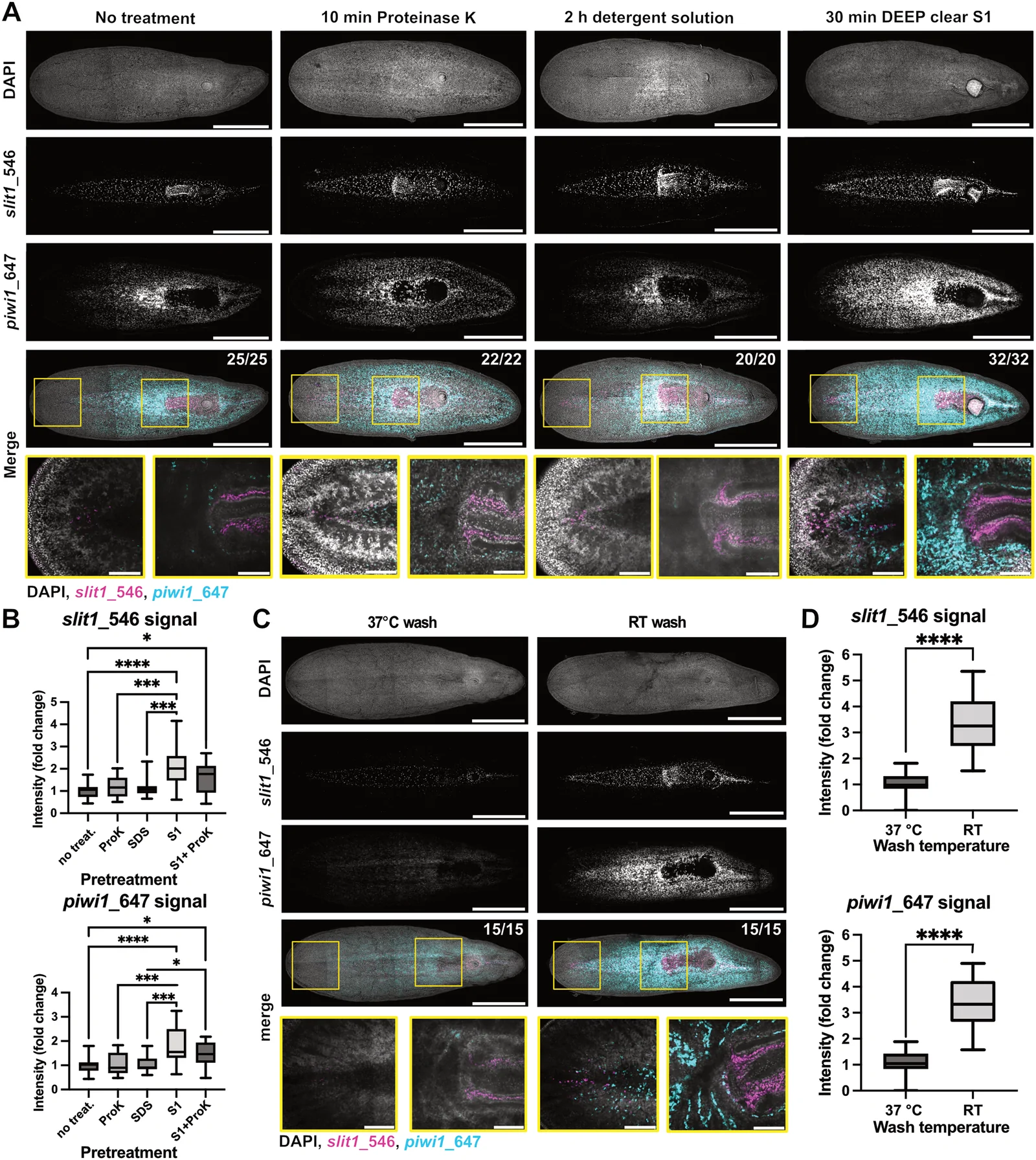
**Optimization of the whole-mount HCR protocol.** (A) Maximum projections of DAPI, *slit1*_546, and *piwi1*_647 signals in entire worms after different tissue permeabilization treatments. Scale bars: 500 µm. The bottom row shows close-ups of single imaging planes in deep tissue layers of the head and the base of the pharynx. Images for 30-min DEEP-Clear S1 are the same as in Fig. S3A (all experimental conditions in both figures were tested in the same experiment). Scale bars: 50 µm. Worms were imaged from the ventral side. Occurrence and total sample sizes per condition are indicated in the merge panels. (B) Quantification of the relative mean signal intensity of *slit1*_546 and *piwi1*_647 after different pretreatments. A total of at least 20 individuals from three independent experiments were analyzed per condition. Boxes represent the values between the 25th percentile and the 75th percentile. Whiskers represent minimal and maximal values (*slit1* signal: *P*<0.0001, *piwi1* signal: *P*<0.0001, ANOVA). no treat.: no treatment, ProK: proteinase K, SDS: SDS detergent solution, S1: DEEP-Clear S1. (C) Maximum projections of DAPI, *slit1*_546, and *piwi1*_647 signals after washing samples at 37°C or RT in HCR wash buffer. Scale bars: 500 µm. The bottom row shows close-ups of single imaging planes in deep tissue layers of the head and the base of the pharynx. Scale bars: 50 µm. Worms were imaged from the ventral side. Occurrence and total sample sizes per condition are indicated in the merge panels. (D) Quantification of the relative mean signal intensity of *slit1*_546 and *piwi1*_647 signals after washing samples at 37°C or RT in HCR wash buffer. A total of at least 15 individuals from two independent experiments were analyzed per condition. Boxes represent the values between the 25th percentile and the 75th percentile. Whiskers represent minimal and maximal values. *slit1* signal: *P*<0.0001, *piwi1* signal: *P*<0.0001, *t*-test; RT: room temperature. *P<0.05, ***P<0.001, ****P<0.0001, ANOVA.

Encouraged by these results, we next explored the utility of HCR for planarian whole-mount staining. As an initial test, we applied the HCR v3.0 protocol developed for whole-mount mouse embryos (Choi et al., 2018) to planarian specimens fixed and permeabilized using the established whole-mount *in situ* hybridization protocol (see Materials and Methods) (King and Newmark, 2013; Pearson et al., 2009). In addition to *piwi1*, we analyzed *slit1* (dd_v6_12111), which is expressed at intermediate levels in multiple cell types associated with the midline, including the pharynx (Cebrià et al., 2007; Reddien et al., 2005) (Fig. 2A). For both probes, we observed the expected patterns, with relatively faint labeling (Fig. S2A). To optimize this signal, we next evaluated the effects of different sample permeabilization methods. Proteinase K (ProK) treatment did improve the HCR signal in deeper tissue layers (Fig. 2A,B), but the peak signal intensity was only slightly increased in *slit1^+^* cells (Fig. 2B). A detergent solution shown to improve signal intensity in a shortened HCR protocol for insects (Bruce et al., 2021) improved neither deep tissue staining nor intensity in our whole-mount samples (Fig. 2A,B). In contrast, DEEP-Clear S1 solution, used as an optional depigmentation step in the DEEP-Clear protocol (Pende et al., 2020), dramatically increased the uniformity of deep tissue labeling and also the peak intensity of the HCR signal in individual cells (Fig. 2A,B). ProK treatment did not further enhance labeling when used in conjunction with DEEP-Clear S1 (Fig. 2B; Fig. S3A). In summary, we find that DEEP-Clear S1 pretreatments yield a pronounced HCR signal increase in planarian whole mounts that makes ProK dispensable.

### Optimization of HCR hybridization and washing steps for planarian whole mounts

Another drawback of classical *in situ* hybridization is the time requirement, especially for multicolor experiments. A single-color FISH protocol takes 4 days, including sample preparation, with every additional target adding another day of processing time (King and Newmark, 2013; Pearson et al., 2009). The HCR v3.0 protocol keeps this process to 4 days, regardless of the number of targets analyzed (Choi et al., 2018). During our studies, we observed that the protocol could be further reduced to 3 days, mainly by shortening the hybridization time from overnight to 2 h without losing noticeable signal intensity (Fig. S4). Importantly, reducing the wash temperature for whole-mount samples from 37°C to 23°C raised the signal intensity by threefold (Fig. 2C,D).

### Compatibility of HCR with different mounting media

**Fig. 3.**
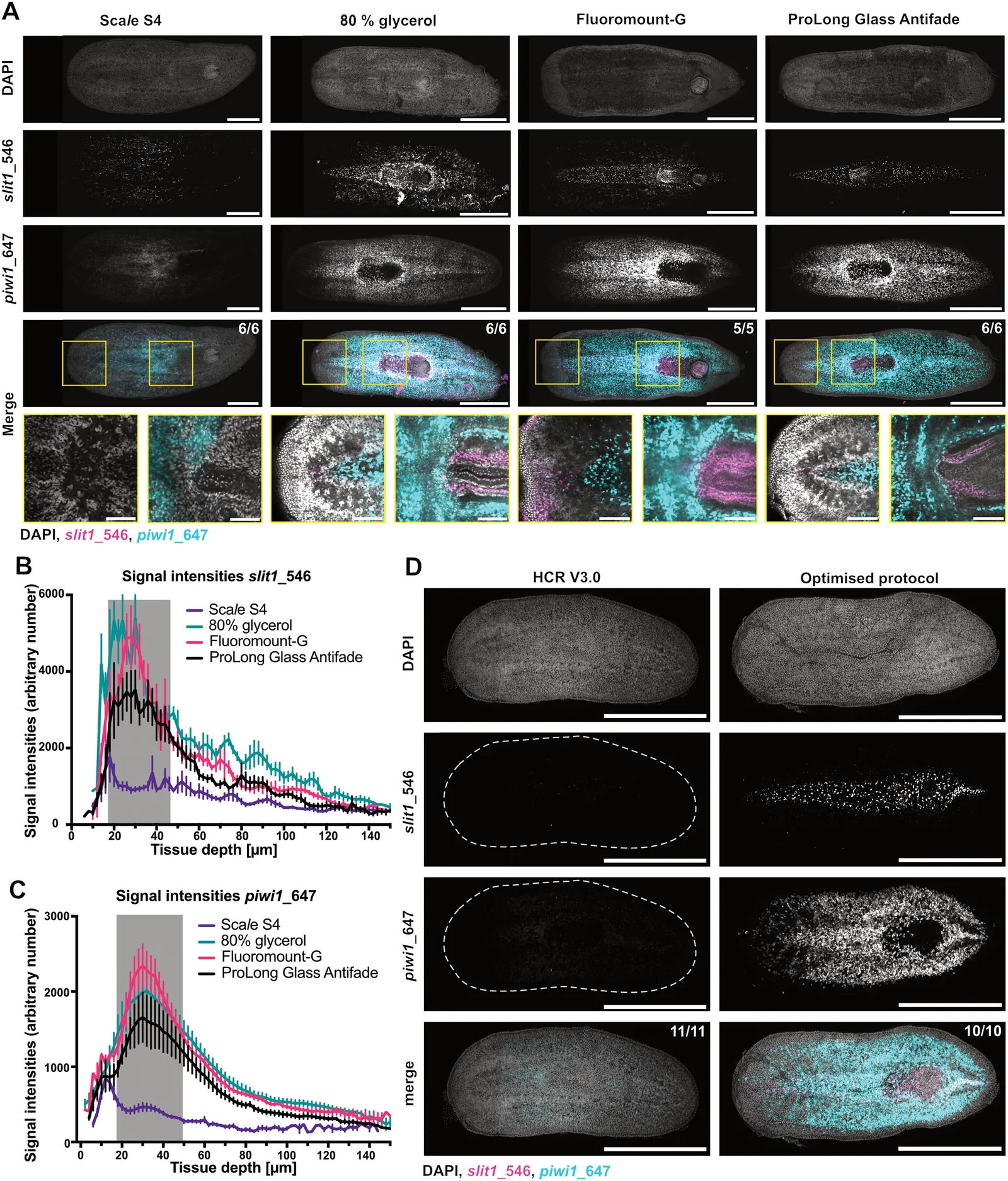
**Influence of mounting media on signal strength in the optimized protocol.** (A) Maximum projections of DAPI, *slit1*_546, and *piwi1*_647 signals of animals mounted in different media. The maximum projection covered the area highlighted in gray in panels B and C. Scale bars: 500 µm. The bottom row shows close-ups of single imaging planes in deep tissue layers of the head and the base of the pharynx. Scale bars: 50 µm. Worms were imaged from the ventral side. Occurrence and total sample sizes per condition are indicated in the merge panels. (B,C) Quantification of the mean *slit1*_546 (B) and *piwi1*_647 (C) signal intensities of samples prepared with different mounting media in comparison to the tissue depth. A total of at least five individuals were analyzed per condition. Error bars denote the standard error of the mean (s.e.m.). (D) Comparison of the original HCR v3.0 protocol and the optimized HCR protocol. Maximum projections of DAPI, *slit1*_546, and *piwi1*_647 signals. Photographs are the same as in Fig. S2A; brightness and contrast adjustments (applied evenly across the compared images within each figure) were optimized for the HCR v3.0 protocol in Fig. S2A and for the optimized protocol here. The settings required to visualize gene expression with the HCR v3.0 protocol result in overexposure of animals labeled with the optimized protocol. Scale bars: 500 µm. Occurrence and total sample sizes per condition are indicated in the merge panels.

We evaluated four different tissue clearing/mounting methods for compatibility with HCR. This step is particularly important in planarians since their tissue opacity can interfere with deep imaging. Sca*l*e S4, a previously established clearing medium for mounting planarian samples for fluorescence microscopy (Hama et al., 2011, 2015), abolished the fluorescence signal with HCR (Fig. 3A). In contrast, using 80% glycerol as a mounting medium preserved the previously described *piwi1* and *slit1* expression patterns. While *slit1*_546 showed the strongest mean signal intensity per cell in glycerol mounts, the brightest signal for *piwi1*_647 was obtained with Fluoromount-G as the mounting medium (Fig. 3B,C). Samples embedded in ProLong Glass Antifade displayed slightly lower signal intensities compared to samples mounted in glycerol or Fluoromount-G, but this was still our medium of choice because it is a hard-set medium that prevents sample drifting during the long imaging times required for whole-mount samples (Fig. 3A-C). We also tried to combine the mounting media with the DEEP-Clear S2 solution that works as a clearing agent in the DEEP-Clear protocol, but the tested experimental conditions did not yield any significant signal intensity improvements (Fig. S3A,B). In summary, we demonstrate that several mounting media are suitable for HCR-processed planarian samples, differing only in resulting signal intensities. The choice of mounting media might therefore be made based on availability, price, microscopy setup and the relative importance of sample immobilization versus maximum signal retrieval. Our final, optimized protocol incorporating the described optimizations (Table 1, see Materials and Methods) substantially increased the signal-to-noise ratio attainable in whole-mount planarians as compared to the original HCR v3.0 protocol (Fig. 3D).

**Table 1.** Protocol timeline

Day	Procedure	Time required
1	1.1 Fixation	0.5 h
	1.2 Bleaching	1.5-2 h
	1.3 Dehydration	Overnight
2	2.1 Rehydration	0.5 h
	2.2 Tissue permeabilization (DEEP-Clear S1)	0.5 h
	2.3 Hybridization	2 h
	2.4 Washing	2.5 h
	2.5 Amplification	Overnight
3	3.1 Washing+post-fixation	3 h
	3.2 Mounting (ProLong Antifade Glass)	2 h curation

A detailed protocol for laboratory use can be found in the Supplementary Materials and Methods.

### HCR robustly detects pattern shifts in planarian whole-mount samples

**Fig. 4.**
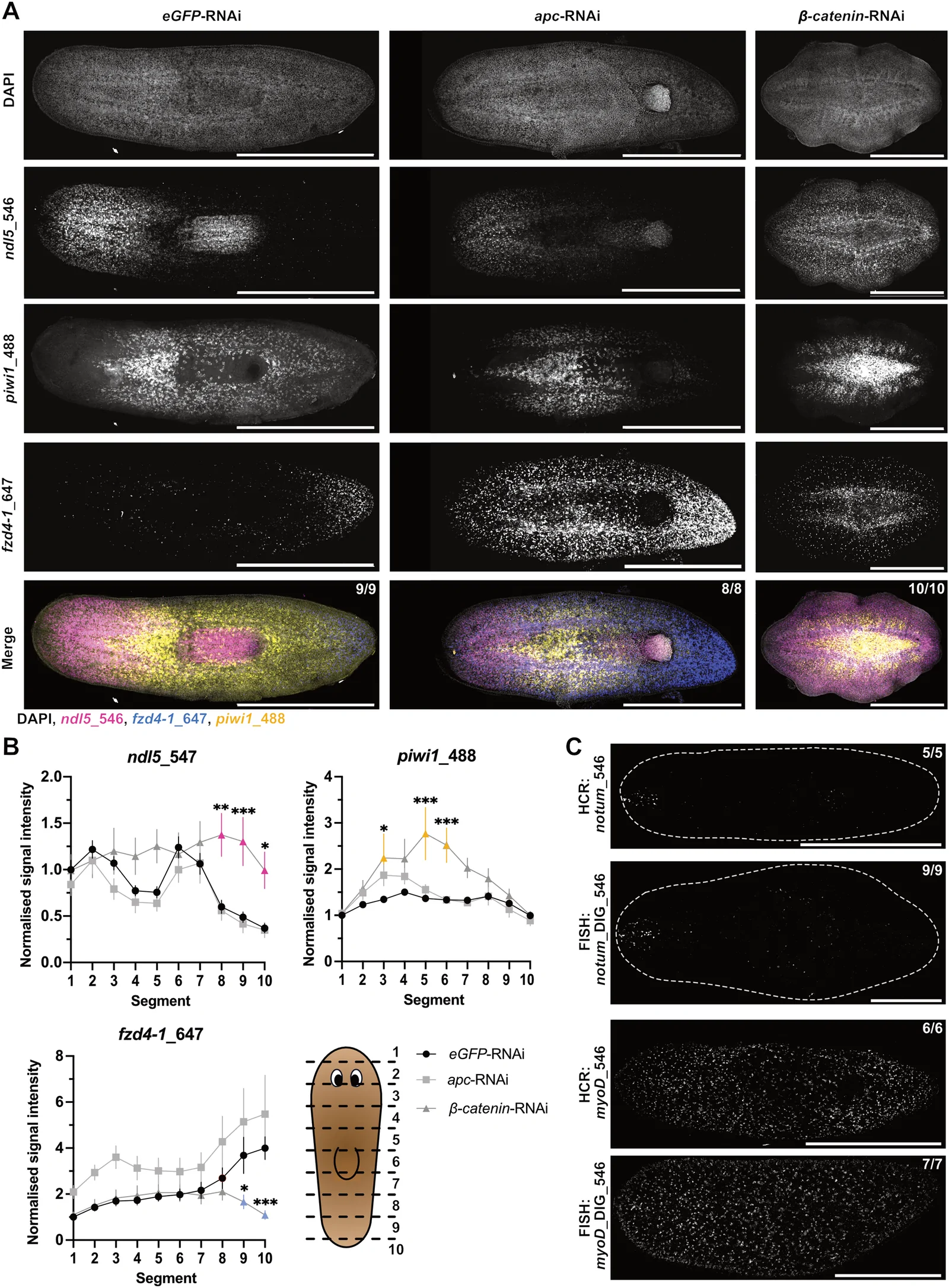
**The optimized HCR in situ hybridization protocol detects pattern shifts in RNAi knockdown animals.** (A) Maximum projections of DAPI, *ndl5*_546, *piwi1*_488 and *fzd4-1*_647 signals. Worms were imaged from the ventral side. Scale bars: 500 µm. Occurrence and total sample sizes per condition are indicated in the merge panels. (B) Average signal intensities of *ndl5*_546, *piwi1*_488 and *fzd4-1*_647 along the anteroposterior axis. A total of at least eight individuals from three independent experiments were analyzed per condition. Error bars denote standard error of the mean (s.e.m.). *slit1* signal: *P*<0.0001, *piwi1* signal: *P*<0.0001, two-way ANOVA. The *P*-values for differences in signal intensity between the control group (*eGFP*-RNAi) and the RNAi knockdowns per body segment were determined by performing Šidák's multiple-comparison test. *P<0.05, **P<0.01, ***P<0.001, ANOVA. (C) Comparison of *notum*_647 and *myoD*_647 expression patterns using FISH and HCR. Scale bars: 500 µm. Occurrence and total sample sizes per condition are indicated in the merge panels.

Finally, we applied our optimized HCR protocol to intact *eGFP-*, *β-catenin-1*- and *apc*-RNAi animals to determine if the dramatic alterations of the planarian body plan can also be visualized with multiplexed HCR (Fig. 4A,B). *eGFP*-RNAi control animals expressed *ndl5* specifically in the head and pharynx, *fzd4-1* specifically in the tail and *piwi1* as described above (Fig. 4A,B), consistent with the known FISH expression patterns of these markers (Reddien et al., 2005; Scimone et al., 2016; Stückemann et al., 2017). *apc* knockdown globally posteriorizes tissue identity (Gurley et al., 2008; Stückemann et al., 2017). As expected, *apc*-RNAi animals showed increased expression of *fzd4-1* throughout the body and reduced expression of *ndl5*, recapitulating the results from our dissociated cell assay at the organismal scale (Fig. 4A,B; Fig. S1). In *β-catenin*-RNAi animals, on the other hand, the known induction of ectopic heads (Adell et al., 2009; Gurley et al., 2008; Petersen and Reddien, 2008) was supported by increased *ndl5* expression and reduced *fzd4-1* expression (Fig. 4A) (Stückemann et al., 2017). To further explore the sensitivity of our improved protocol, we also targeted genes with low expression in intact animals, like *notum* (dd_v6_24180) and *myoD* (dd_v6_12634). Both genes could be detected with our optimized HCR protocol, demonstrating that it has comparable sensitivity to FISH (Fig. 4C). Taken together, these data demonstrate that our adapted HCR protocol is suitable for both visualizing and quantifying changes in mRNA expression patterns in an accurate, fast, sensitive and multiplexed manner.

To test the applicability of our protocol to other planarian species, we also labeled the marine planarian *Camerata robusta* (*Crob*; Vila-Farré et al., 2023), which to our knowledge, has not been subject to *in situ* hybridization to date. For *Crob-slit1*, a stripe-like pattern along the midline was observed that, while less intense, is similar to the pattern in *S. mediterranea* (Fig. S2B). For *Crob-piwi1*, expression could be detected throughout the entire body, with increased expression around the testis (Fig. S2B). That our optimized protocol also performs well in this species (Fig. S2B) confirms the robustness and versatility of our approach. In conclusion, the HCR protocol reported here simultaneously improves both the technical capability and convenience of analyzing multiplexed gene expression in planarians, making it a valuable addition to the tool kit for studying these remarkable model organisms.

### Conclusion

In this study, we established a robust and sensitive HCR protocol for whole-mount planarians and cell preparations of dissociated worms. Our protocol reduces the time requirement of *in situ* hybridization to 3 days, independent of the number of targets. The optimized protocol allows the user to detect RNA expression at the cellular level throughout different tissue layers with a high signal-to-noise ratio, providing a valuable resource for multiplexed gene expression analysis in a model system for tissue regeneration.

## MATERIALS AND METHODS

### Planarian maintenance

Experiments with *S. mediterranea* used the asexual (CIW4) strain (Sánchez Alvarado et al., 2002). The animals were cultured in 1× Montjuic salts [1.6 mmol l^−1^ NaCl, 1.0 mmol l^−1^ CaCl_2_, 1.0 mmol l^−1^ MgSO_4_, 0.1 mmol l^−1^ MgCl_2_, 0.1 mmol l^−1^ KCl and 1.2 mmol l^−1^ NaHCO_3_, in double-distilled water (ddH_2_O) water] at 20°C in aquaria (Vu et al., 2019). At least 3 weeks before experiments, animals were transferred into plastic boxes, treated with ciprofloxacin (100 µg ml^−1^), fed with homogenized calf liver, and amputated to create multiple fragments. Animals were then allowed to regenerate for 14 days before use. Experiments with *C. robusta* used the laboratory strain of the Rink Department (Vila-Farré et al., 2010), cultured in plastic boxes with marine water (16‰ sea salt in Milli-Q water) at 20°C. Animals were fed with homogenized calf liver and proliferated by sexual reproduction. Only fully matured worms were used in the experiments.

### Irradiation

Worms were exposed to a 60 Gy dose of gamma radiation using a CellRad irradiation device (Precision X-Ray, North Branford, CT, USA) and macerated either 1 or 6 dpi. Cell preparations from nonirradiated animals were used as a control.

### RNAi experiments

Double-stranded RNA (dsRNA) corresponding to *eGFP*, *β-catenin* (dd_v6_2688) and *apc* (dd_v6_6966) was generated by amplifying target genes from *S. mediterranea* cDNA, using gene-specific primers (Table S4) and then inserting the PCR product into the pPR-T4P plasmid via Gibson Assembly (Gurley et al., 2008). For dsRNA synthesis, the target genes were amplified from the plasmids by performing PCRs with primers that add a T7 promoter to both ends of the target gene. For subsequent *in vitro* transcriptions, 4 µg of DNA template was mixed with 1× *in vitro* transcription buffer [25 mM MgCl_2_, 40 mM Tris pH 8.0, 10 mM dithiothreitol (DTT), 2 mM spermidine], 5% DMSO, 1 U µl^−1^ RNase inhibitor (Thermo Fisher Scientific), 0.0015 U µl^−1^ inorganic pyrophosphatase (Thermo Fisher Scientific), 25 mM rNTPs (Thermo Fisher Scientific) and 3.4 U µl^−1^ T7 polymerase (ribonucleotide triphosphates) (Thermo Fisher Scientific) to a final volume of 200 µl. The reaction was then incubated at 37°C overnight. The reaction was then heated to 95°C for 3 min at 700 rpm on the shaker. After cooling down, the dsRNA was purified by mixing the reaction with one volume of NaCl/PEG-8000 precipitation solution. After centrifugation, the dsRNA pellet was washed in 70% ethanol and then resuspended in RNase-free water. For feeding, the dsRNA was mixed 1:1 (v/v) with organic calf liver at a final concentration of 1 µg µl^−1^ (Rouhana et al., 2013). Worms were fed once every 3 days for three total feedings over 6 days and then starved for 8 days before fixation. RNAi worms were kept in a flow system with planarian water (Cebrià and Newmark, 2005).

### Tissue preparation for dissociated cells

To obtain single-cell suspensions, we dissociated three worms per condition in 1 ml of ACME solution (García-Castro et al., 2021). 10% (v/v) DMSO was then added as cryoprotectant prior to storing samples at −20°C (Reddien et al., 2005).

### HCR probe synthesis

HCR probes (Tables S1 and S2) were generated using the HCR *in situ* probe generator from the Özpolat Lab (Kuehn et al., 2022). HCR probe pools are listed in Dataset 1.

### HCR RNA-FISH on dissociated cells

Dissociated cells were treated according to the HCR v3.0 protocol for mammalian cells in suspension (Choi et al., 2018), with several alterations. Briefly, 60,000 cells per well were plated in a flat-bottom 96-well plate (Greiner Bio-One, 655891). After centrifugation at 1000 ***g*** for 5 min at 4°C, the cells were incubated in 70% (v/v) ethanol overnight. After prehybridization the next day, 0.4 µl of each probe was added per well in 50 µl of probe hybridization buffer and incubated for 2 h at 37°C. Afterward, the probes were washed out at room temperature (RT, 23°C) prior to pre-amplification and overnight incubation at RT with hairpins. On the last day of the procedure, unbound hairpins were washed out, and the cells were mounted in 50 µl of SlowFade mounting medium with DAPI. Images were analyzed with scanR v3.5 software (Evident). First, the raw fluorescence images of the nuclear (DAPI) channel were segmented using the ‘edge’ module, which accounts for variation in nuclear staining intensities. Nuclei masks were then geometrically enlarged, filtered according to the nuclei's maximum and mean intensity to eliminate doublets and debris (Fig. 1A), and used to analyze the fluorescence intensity of each cell in each particular channel under the enlarged mask. Within these gated nucleated cells, we set the gate for positive cells so that npc showed a maximum positivity of 0.1% in each channel (Fig. S1).

### HCR whole-mount sample preparation

Before fixation, 2-4 mm worms were transferred into 15 ml Falcon tubes. Planarian water was replaced with pH-neutralized *N*-acetylcysteine (0.5% NAC). Subsequently, the animals were euthanized using 5% NAC for 5 min, fixed in 4% paraformaldehyde (PFA) in 0.5× phosphate buffered saline with 0.3% Triton X-100 (PBSTx0.3) for 30 min, treated with 0.125 M glycine in 1× PBSTx0.3 for 10 min, and washed in 1× PBSTx0.3. Worms were then treated with a DTT-containing reduction solution for 10 min, washed in 1× PBSTx0.3, dehydrated with 50% methanol followed by 100% methanol in 1× PBSTx0.3, and stored overnight at −20°C in 100% methanol (Pearson et al., 2009). Specimens were rehydrated and then bleached for 1.5-2 h using formamide bleach [5% non-deionized formamide, 1.2% H_2_O_2_, in 0.5× saline-sodium citrate buffer (SSC)] until they turned entirely white (King and Newmark, 2013). Bleached worms were treated with DEEP-Clear S1 solution (Pende et al., 2020) for 30 min at 37°C and washed in 5× SSCT (SSC with 0.1% Triton X-100). Tissue preparation is explained in detail in the Supplementary Materials and Methods.

### HCR RNA-FISH in whole mounts

Worms were prehybridized in HCR hybridization buffer for 30 min at 37°C, hybridized for 2 h at 37°C in HCR hybridization buffer with probes for the relevant transcripts (10 nM final concentration), and then washed four times for 30 min each in HCR wash buffer at RT. Worms were subsequently rinsed with 5× SSCT and pre-amplified for 10-20 min in HCR amplification buffer. For amplification, hairpins were added to the HCR amplification buffer at a final concentration of 60 nM per hairpin with DAPI at a final concentration of 1 ng µl^−1^. Worms were incubated overnight in this solution at RT, washed in 5× SSCT for over 1 h, and then post-fixed for 10 min in 4% PFA. Finally, worms were washed with 0.125 M glycine in 5× SSCT for 10 min, washed in 5× SSCT, and mounted on glass coverslips with 100 or 250 µm circular adhesive spacers. 5× SSCT was replaced with ProLong Antifade Glass Mounting medium (Thermo Fisher Scientific, P36980), and a second coverslip was used to seal the animals.

### Cloning and riboprobe synthesis

Target genes for FISH were amplified from *S. mediterranea* cDNA, using gene-specific primers (Table S3, HCR probe pools and riboprobes list) and then via Gibson Assembly inserted in the pPR-T4P plasmid (Gurley et al., 2008). Antisense riboprobes were synthesized from those constructs by amplifying the genes via PCR using the primers cattaccatcccgTTAATTGAGTGAGATGTGCC and ccaattctacccgTCGACGATCACATAACTAACC for the *notum*-DIG-rhodamine probe and the primers cattaccatcccgTTTCCAGGTTCCACTTGTCC and ccaattctacccgCAATATGTATCAGGGCAAACG for the *myoD*-DIG-rhodamine probe, followed by *in vitro* transcription. For *in vitro* transcription, 1 µg of DNA template was mixed with 1× *in vitro* transcription buffer (25 mM MgCl_2_, 40 mM Tris pH 8.0, 10 mM DTT, 2 mM spermidine), 5% DMSO, 1 U µl^−1^ RNase inhibitor (Thermo Fisher Scientific), 0.0015 U µl^−1^ inorganic pyrophosphatase (Thermo Fisher Scientific), 1× DIG RNA labeling mix (Roche) and 2 U µl^−1^ T7 polymerase (Thermo Fisher Scientific). The mixture was incubated overnight at 37°C. The riboprobe was precipitated using 10 ml of 7.5 mM ammonium acetate and 50 µl ice-cold 100% ethanol. After centrifugation, the pellets were washed in 75% ice-cold ethanol and then resuspended in deionized formamide (Lee et al., 2024).

### FISH of whole-mount samples

Whole-mount FISH samples were prepared as described for HCR, but bleached worms were treated with ProK solution [0.1% SDS, 2 µg µl^−1^ ProK (NEB), 1× PBS] for 10 min (Pearson et al., 2009). Worms were then placed in 4% PFA in 1× PBSTx0.3 for 10 min at RT. After being rinsed twice with 1× PBSTx0.3, worms were washed in 1:1 1× PBSTx0.3:PreHybridization buffer for 10 min at RT. Afterward, the worms were incubated in PreHybebridization buffer (50% formamide, 5× SSC, 1× Denhardt's solution, 100 µg µl^−1^ heparin, 1% Tween 20, 1 mg ml^−1^ Roche torula yeast RNA, 50 mM DTT in ddH_2_O) for 2 h at 58°C. The riboprobes (1:2000) were denatured in hybridization buffer (50% formamide, 5× SSC, 1× Denhardt’s solution, 100 µg µl^−1^ heparin, 1% Tween 20, 0.25 mg ml^−1^ Calbiochem yeast RNA, 5% dextran sulfate, 50 mM DTT in ddH_2_O) for 3 min at 70°C. PreHybridization buffer was replaced with the riboprobe mixture and incubated for 16 h at 58°C (Pearson et al., 2009). Worms were then washed two times for 20-30 min in WashHybridization buffer (50% formamide, 0.5% Tween 20, 1× Denhardt's solution in ddH_2_O) at 58°C, followed by two times for 20-30 min in 1:1 2× SSCTw0.1 (2× SSC with 0.1% Tween 20):WashHybridization buffer at 58°C, three times for 20-30 min in 2× SSCTw0.1 at 58°C, three times for 20-30 min in 0.2× SSCTw0.1 (0.22× SSC with 0.1% Tween 20) at 58°C and three times for 10 min in PBSTx0.3 at RT (Pearson et al., 2009). Worms were blocked for 2 h using 5% sterile horse serum and 0.5% Roche Western Blocking reagent in PBSTx0.3. The DIG-antibody (1:2000, Roche, 11207733910) and DAPI (1:3000) incubation was then done in 5% sterile horse serum and 0.5% Roche Western Blocking reagent in PBSTx0.3 at 4°C overnight. After antibody incubation, worms were washed six times for 20 min in PBSTx0.3. Preincubation before development was done for 15 min in TSA buffer (2 M NaCl, 0.1 M boric acid pH 8.5 in ddH_2_O) with rhodamine-tyramide (1:2000) and 4-(*p*-iodophenyl) butyric acid (1:1000 of 20 µg µl^−1^ solution). Development was induced by adding 30% H_2_O_2_ (1:5000) to the solution and incubating the worms for 45 min at RT. After development, samples were washed two times for 10 min in PBSTx0.3. PBSTx0.3 was then replaced with Sca*l*e S4 (Hama et al., 2015). Worms were incubated overnight and then mounted in this solution as described above.

### Image acquisition

For microscopy, an Olympus IX83 confocal microscope equipped with a Yokogawa CSUW1-T2S spinning disk scan head and a Hamamatsu Orca Flash 4.0 v3 monochrome scientific complementary metal-oxide-semiconductor (sCMOS) camera (2048×2048) was used. Animals were imaged using an Olympus 20× UPlanXApo NA 0.8 objective. All channels were imaged sequentially. DAPI was excited with a 405 nm laser, and the signal was captured with a 447/50 bandpass filter. Probe *piwi1*_488 was excited with a 488 nm laser, and the signal was captured with a 525/50 bandpass filter. Probes *slit1_*546, *notum*_546, *myoD*_546 and *ndl5_*546, as well as *notum*-DIG-rhodamine and *myoD*-DIG-rhodamine, were excited with a 560 nm laser, and the signal was captured with a 595/50 bandpass filter. Probes *piwi1_*647 and *fzd4-1_*647 were excited with a 640 nm laser, and the signal was captured with a 685/40 bandpass filter.

### Image analysis and quantification of whole-mount images

Microscopy images were processed and analyzed using Fiji version 2.14/1.54f (Schindelin et al., 2012). Z-stacks were projected using the ‘maximum intensity projection’ module. Mean signal intensities were quantified for objects 30-5000 pixels in size with a circularity factor of between 0.5 and 1.0 using the standard ‘Analyze particles’ tool. For thresholding maximum projections, the automatic threshold ‘IsoData’ was used to detect *piwi^+^* cells, and ‘Moments’ thresholding was used to identify *slit1^+^* cells. For thresholding single-plane recordings, the automatic threshold ‘Moments’ was used to identify *piwi^+^* cells, and ‘RenyiEntropy’ was used for detecting *slit1^+^* cells. All automatic thresholds were run with the ‘dark background’ setting. To quantify signal intensities along the anteroposterior axis, maximum projected image stacks were down-sampled (bicubic), along the animal's mediolateral axis, to 0.1% of their initial width to bin intensity values. Subsequently, the line tool was used to manually mark the anteroposterior axis, and the signal intensities along this line were recorded using the ‘Plot Profile’ function. Data were then binned into ten equal segments along the A/P axis. Values of every bin were averaged to obtain the final intensity values plotted in the graphs.

### Statistical analysis

Creation of graphs and statistical analysis was performed with GraphPad Prism 10.4.1. For comparisons of two conditions, unpaired *t*-tests with Welch's correction were performed. For comparisons of multiple conditions, analyses of variance (ANOVAs) with multiple comparisons were performed. For the pretreatment dataset and the hybridization time course dataset, Brown-Forsythe ANOVAs and Welch's ANOVAs were performed with Dunnett's T3 multiple-comparison tests to compare all pretreatment methods to each other. For the three-color analysis, two-way ANOVAs with Šidák’s multiple comparisons were performed to compare the RNAi conditions in a body segment to the control group (*eGFP*-RNAi) of the body segment.

## Supplementary Material



10.1242/biolopen.062669_sup1 Supplementary information

Dataset 1. HCR probe pools and riboprobes list.
