# Supplementary material for: Multiplexed gene expression analysis in dissociated and intact planarian flatworm tissues using in situ hybridization chain reaction
Source: Biol Open. 2026 Sep 10;15(9):bio062669. doi: 10.1242/bio.062669 (PMC13602376; doi:10.1242/bio.062669)
Supplement: Supplementary file 1 [file biolopen-15-062669-s1.pdf]

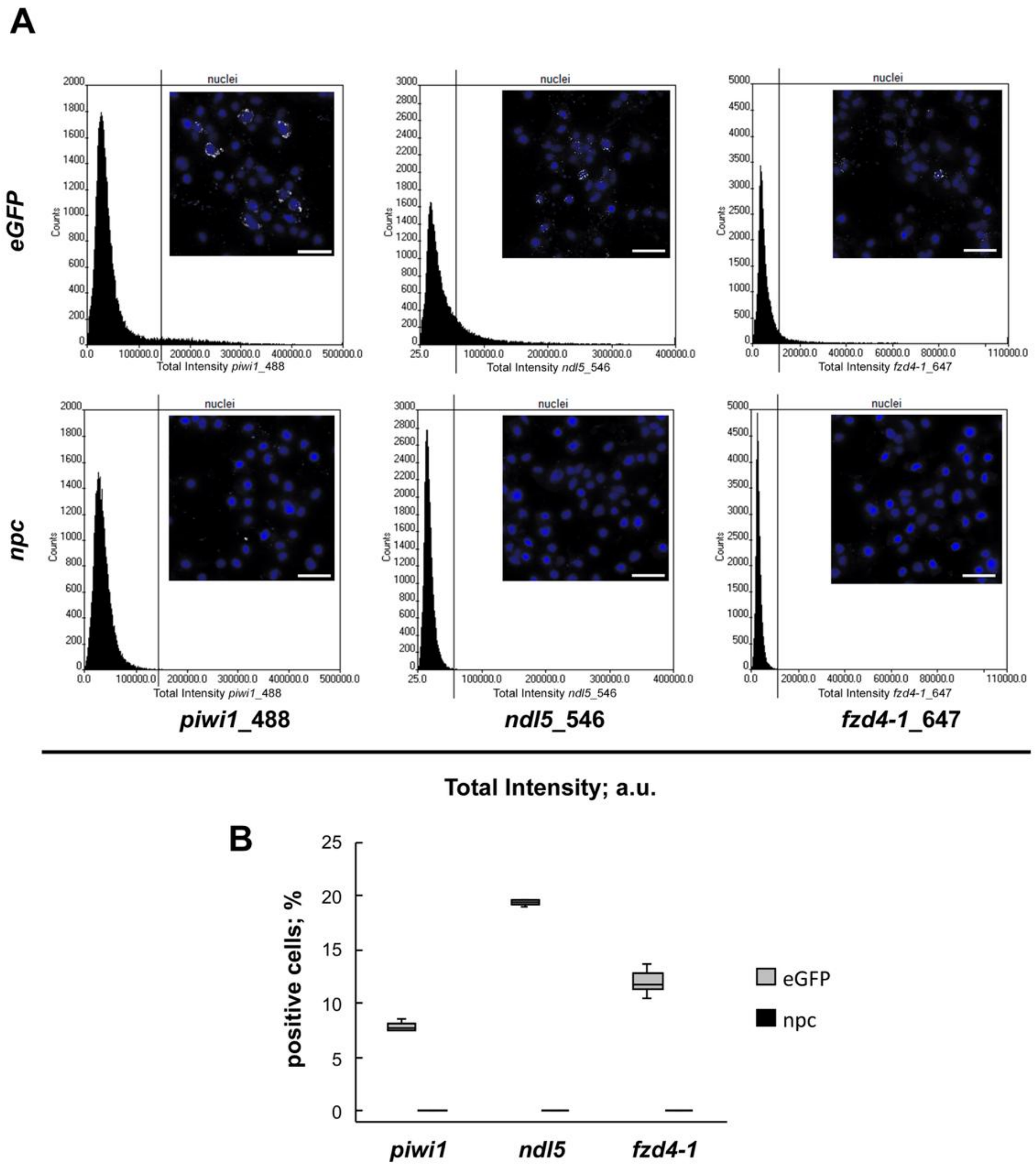

Fig. S1. Negative controls for HCR on planarian dissociated cells. (**A**) Histograms of dissociated cells labelled with HCR probes specific for *piwi1*_488, *ndl5*_546, and *fzd4- 1*_640. npc cells were treated only with fluorescently labelled hairpins (i.e., no HCR probes were applied). Insets show representative images; HCR signal in gray, DAPI in blue. Histograms and insets for eGFP-RNAi are the same as in Figure 1D (all RNAi conditions in both figures were tested in the same experiment). Scale bars: 20 µm. (**B**) Quantification of cells positive for *piwi1*, *ndl5*, and *fzd4-1*. Data are from 5 replicates of 10 animals each. Boxes represent the values between the 25th percentile and the 75th percentile. Whiskers represent minimal and maximal values.

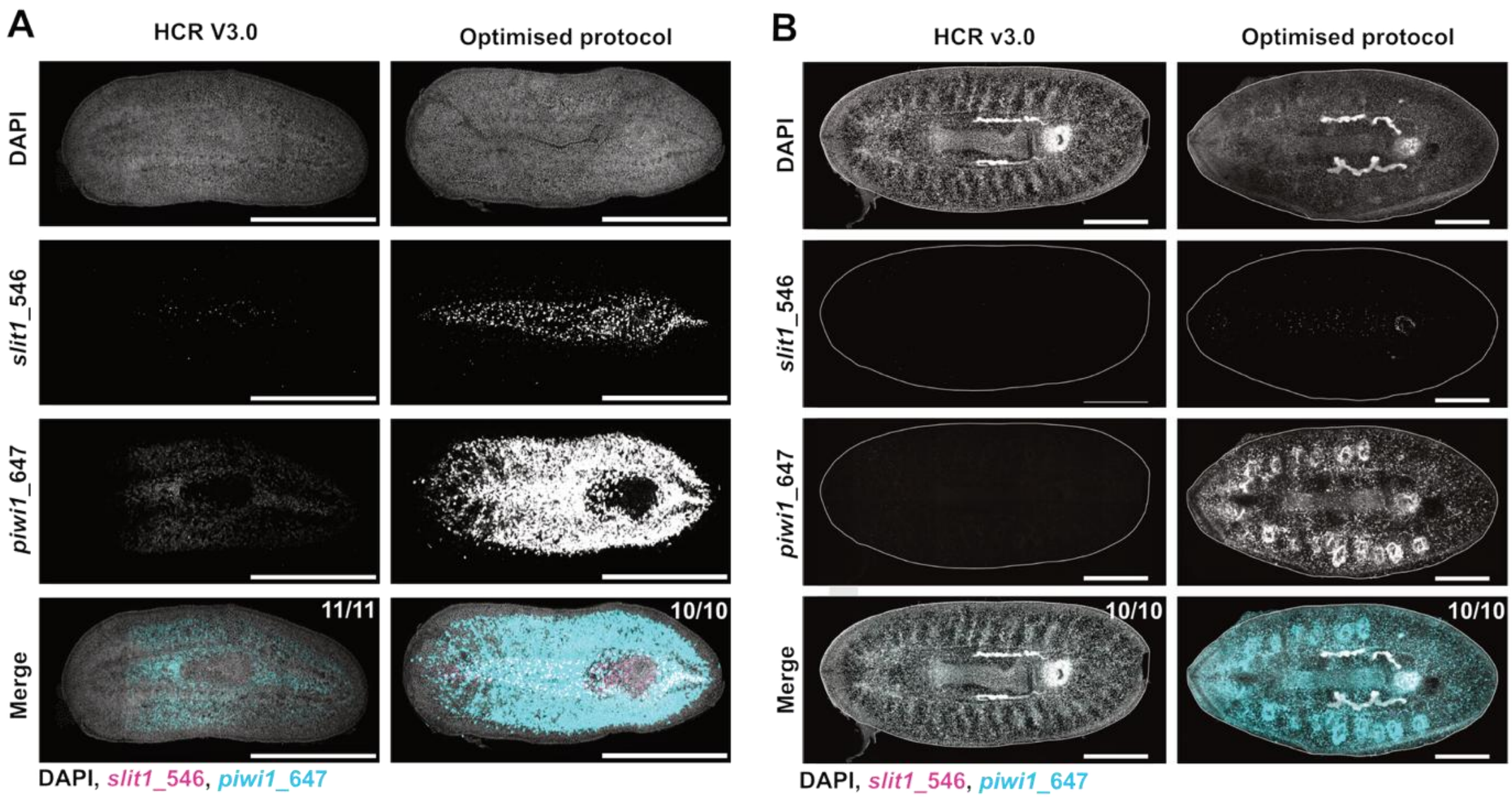

Fig. S2. Comparison between the original HCR v3.0 protocol and the optimised HCR protocol. **(A)** Representative maximum projections of DAPI, *slit1*_546, and *piwi1*_647 in *Schmidtea mediterranea*. Photographs are the same as in Figure 3D; brightness and contrast adjustments (applied evenly across the compared images within each figure) were optimised for the HCRV3.0 protocol here and for the optimised protocol in Figure 3D. The settings required to visualize gene expression with the HCRV3.0 protocol result in overexposure of animals labelled with the optimised protocol. Scale bars: 500 µm. Occurrence and total sample sizes per condition are indicated in the merge panels. **(B)** Representative maximum projections of DAPI, *slit1*_546, and *piwi1*_647 in *Camerata robusta*. Scale bars: 500 µm. All worms were imaged from the ventral side. Occurrence and total sample sizes per condition are indicated in the merge panels.

Biology Open • Supplementary information

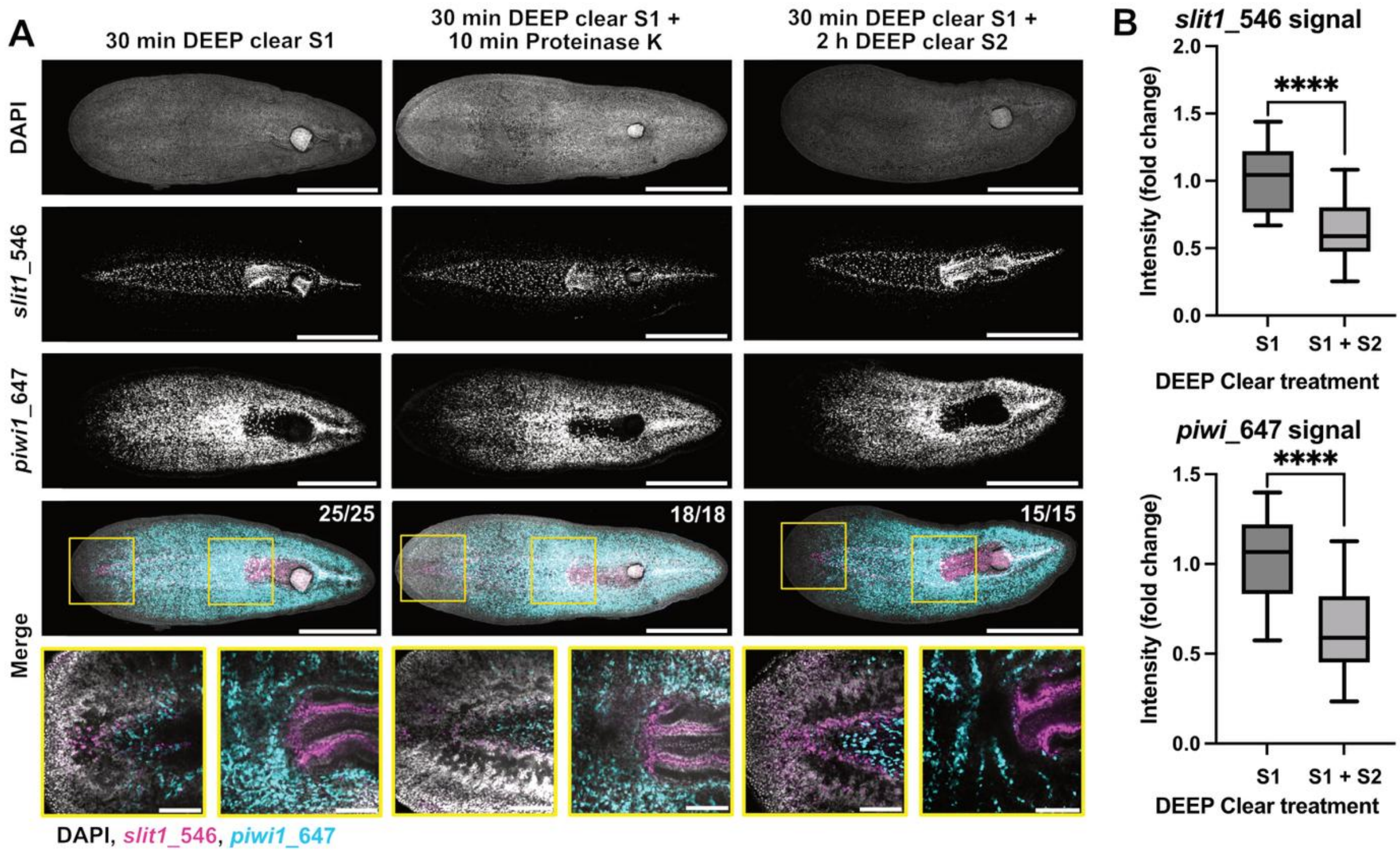

Fig. S3. Impact of Proteinase K and DEEP Clear S2 on signal intensity in the optimized protocol. (**A**) Maximum projections of DAPI, *slit1*_546, and *piwi1*_647 signal in entire worms after different tissue permeabilization and tissue clearing treatments. Scale bars: 500 µm. The bottom row shows close-ups of single imaging planes in deep tissue layers of the head and the base of the pharynx. Images for 30 min DEEP clear S1 are the same as in Figure 2A (all experimental conditions in both figures were tested in the same experiment). Scale bars: 50 µm. Worms were imaged from the ventral side. Occurrence and total sample sizes per condition are indicated in the merge panels. (**B**) Quantification of the relative mean *slit1*_546- and *piwi1*_647 signal intensity of samples with and without DEEP Clear S2 treatment. A total of at least 15 individuals from 2 independent experiments were analysed per condition. Boxes represent the values between the 25th percentile and the 75th percentile. Whiskers represent minimal and maximal values. (*slit1* signal: *P* value < 0.0001, *piwi1* signal: *P* value < 0.0001) t tests.

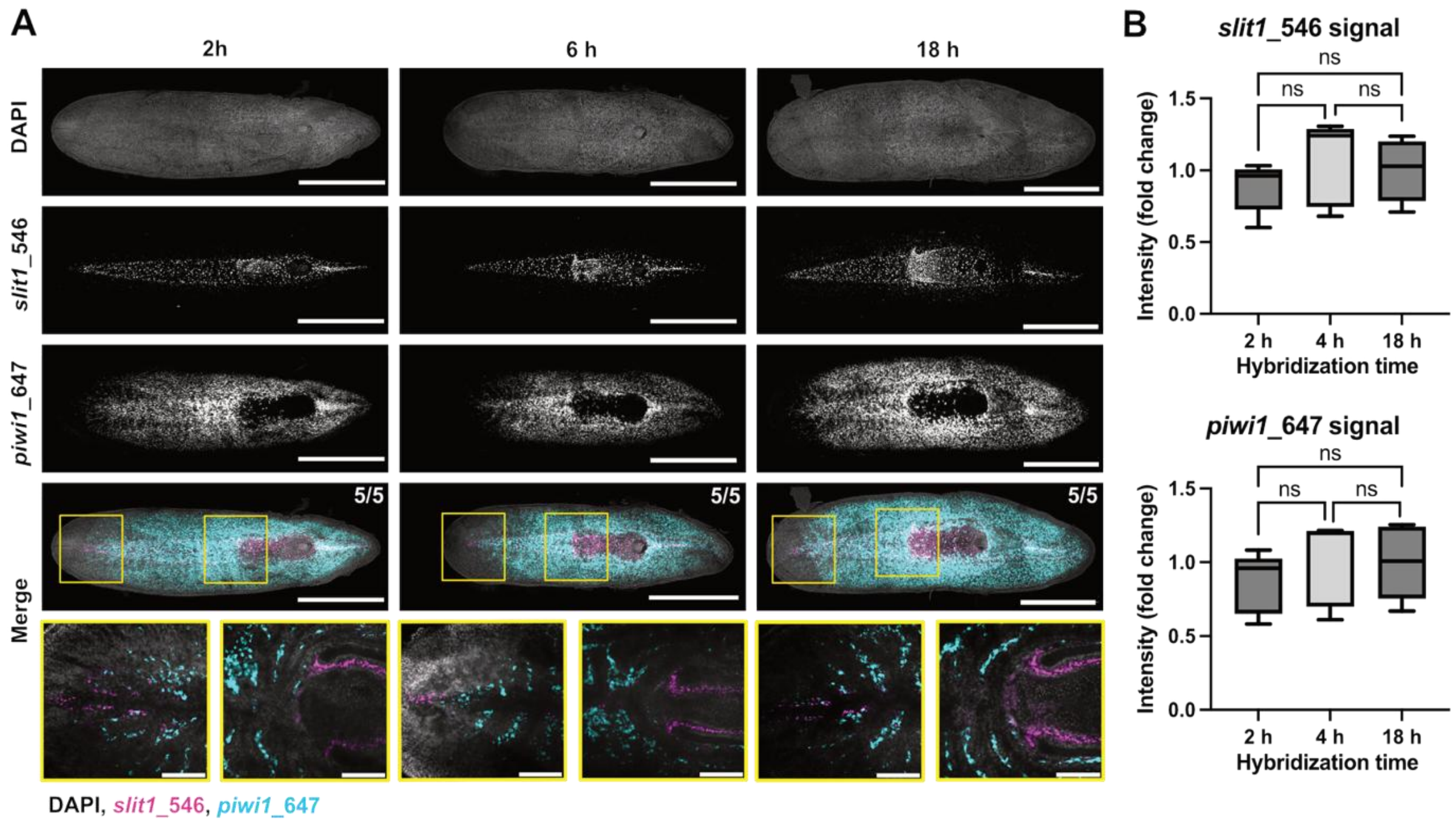

Fig. S4. Impact of hybridization time on signal intensity in the optimized protocol. (**A**) Maximum projections of DAPI, *slit1*_546, and *piwi1*_647 signal in entire worms after different hybridization times. Scale bars: 500 µm. The bottom row shows close-ups of single imaging planes in deep tissue layers of the head and the base of the pharynx. Scale bars: 50 µm. Worms were imaged from the ventral side. Occurrence and total sample sizes per condition are indicated in the merge panels. (**B**) Quantification of the relative mean *slit1*_546- and *piwi1*_647 signal intensity after different hybridization times. A total of at least 5 individuals were analysed per condition. Boxes represent the values between the 25th percentile and the 75th percentile. Whiskers represent minimal and maximal values. (*slit1* signal: *P* value = 0.5027, *piwi1* signal: *P* value = 0.6092) one-way ANOVA; ns: non-significant.

Dataset 1. HCR probe pools and riboprobes list.

Available for download at
https://journals.biologists.com/bio/article-lookup/doi/10.1242/bio.062669#supplementary-data

Biology Open • Supplementary information

Table S1. HCR probes – *Schmidtea mediterranea*

Probe name	Probe adaptor	Transcript ID
*fzd4-1*_647	B3	dd_Smed_v6_11650_0_1
*ndl5*_546	B1	dd_Smed_v6_5102_0_1
*notum*_546	B1	dd_Smed_v6_24180_0_1
*myoD*_546	B1	dd_Smed_v6_12634_0_1
*piwi1*_488	B2	dd_Smed_v6_659_0_1
*piwi1*_546	B1	dd_Smed_v6_659_0_1
*piwi1*_647	B3	dd_Smed_v6_659_0_1
*slit1*_546	B1	dd_Smed_v6_12111_0_1

Table S2. HCR probes – *Camerata robusta*

Probe name	Probe adaptor	Transcript ID
*piwi1*_647	B2	dd_Came_v1_15226_1_1
*slit1*_546	B3	dd_Came_v1_8268_1_1

Sequences of the HCR probes can be found in the HCR probe pools and Riboprobes list.

Table S3. WISH riboprobes

Probe name	Transcript ID
notum-DIG	dd_Smed_v6_24180_0_1
myoD-DIG	dd_Smed_v6_12634_0_1

Sequences of the riboprobes and primers used for cloning can be found in the supplementary excel sheet.

Table S4. dsRNA for RNAi

Gene name	Transcript ID
eGFP	synthetic
β-catenin-1	dd_Smed_v6_2688_0_1
apc	dd_Smed_v6_6966_0_1

Sequences of the dsRNA and the primers used for cloning can be found in the supplementary excel sheet.

Biology Open • Supplementary information

Supplementary Materials and Methods

Detailed bench protocols

HCR protocol for whole-mount planarians

This protocol was modified form the protocol for chicken embryos from Choi *et al*. (Choi et al., 2018).

1. **Preparation**

   1.1 - Starve worms for at least two weeks before fixing to reduce the background in the intestines.

2. **Fixation**

   Notes:

   - This protocol is designed for up to 50x 2-5 mm worms. For bigger worms, use longer times for mucus removal, fixation and reduction.

   2.1 - Transfer planarians to a 15 ml conical tube in planarian water.

   2.2 - Replace planarian water with 13 ml pH-neutralised NAC (0.5%), and incubate for 10 min at RT on a tube roller.

   2.3 - Replace pH-neutralised NAC with 13 ml 5% NAC in 1x PBSTx0.3 solution, and incubate for 5 min at RT on the tube roller.

   Notes:

   -NAC is a mucolytic reagent. Increase time/concentration for bigger worms.

   - In general:
   - For 3-5 mm worms, 5% NAC and 5 min
   - For 5-8 mm worms, 7.5% NAC for 5 min
   - For > 8mm, 7.5% NAC for 10 min or 10% NAC for 5 min
   - NEVER prepare NAC in H_2O; otherwise, the worms will dissolve!

   2.4 - Remove NAC, but leave a little residual liquid to cover the worms and avoid air contact.

   2.5 - Add 2 ml PFA-fixation solution.

   2.6 - Replace the solution with 5 ml of 4% PFA-fixation solution, close the cap of the tube, and rinse the samples by carefully tilting the tube.

Biology Open • Supplementary information

2.7 - Replace the solution with roughly 10 ml of 4% PFA solution. Avoid air contact with the worms. Lay the tubes flat on the bench, and fix for 30 min at RT with occasional rocking every 5-10 min.

Note: For worms that are bigger than 5 mm, fix for more than 60 min at RT.

2.8 - Remove the fixative, and rinse the worms 1x 10-15 min with 0.125M glycine in PBSTx0.3 to reduce autofluorescence.

2.9 - Rinse worms 2x 5-10 min with 1X PBSTx0.3.

3. Reduction

3.1 - Replace PBSTx0.3 with pre-heated reduction solution (add DTT to the reduction solution directly before use), and incubate at 37 °C for 5 min

Note: for bigger worms, incubate for 10 min (6-10mm) or 15 min (>10mm).

3.2 - Remove the reduction solution, and wash the worms 2x 10 min with 1x PBSTx0.3, rocking at RT.

Note: either bleach at this step, dehydrate and store the worms in MeOH until HCR, or dehydrate the worms now and bleach directly before the permeabilization step.

4. Formamide bleach

- Bleach worms either after fixation and reduction and directly before dehydration or after rehydration and before permeabilization.

4.1 - Rinse worms with 1x SSC, 5–10 min with rocking at RT.

4.2 - Replace 1x SSC with formamide bleaching solution. Bleach on a light table, 1.5-2 h).

Notes:

- It is not recommended to bleach 2-4 mm worms more than 2 hr in formamide because it might damage the *in-situ* signal.
- To prepare the bleaching solution, add formamide and H_2O_2 into 0.5x SSC. NEVER add H_2O_2 directly to formamide (danger of explosion).

4.3 - Wash the worms in 1x SSC for 5 - 10 min, rocking at RT.

4.4 - Wash worms 2x with 1x PBSTx0.3 for 5-10 min, rocking at RT.

Biology Open • Supplementary information

5. Dehydration

5.1 - Replace 1x PBSTx0.3 with 50% Methanol solution for 5–10 min, rocking at RT.

5.2 - Replace 50% Methanol solution with 100% Methanol, 2x 5 min, rocking at RT.

5.3 - Store the sample at -20 °C for at least for 1h or ON.

6. Rehydration

6.1 - Replace 100% Methanol with 75% and then 50% Methanol solution, for 5–10 min each, rocking at RT.

6.2 - Replace 50% Methanol solution with 1x PBSTx0.3 for 5–10 min, rocking at RT.

6.3 - Wash worms 2x with 1x PBSTx0.3 for 5–10 min, rocking at RT.

7. Tissue permeabilization

7.1 - Transfer up to 12 worms per sample into a sieve bucket and put the bucket into a 96-well plate. Unless stated otherwise, use 180 µl solution per well in each of the following steps.

Notes:

- The buckets can be made from PCR tubes. Cut off the lower half or the tube, heat it slightly on a heating plate and use the slightly melted plastic to glue 20 µm nylon netting to it.
- 24 well plates work in principle with 600 µl solution, but this is more expensive when it comes to the usage of the hairpins.

7.2 - Incubate samples for 30 min in DEEP clear solution S1 at 37 °C.

7.3 - Wash the worms 2 x 5 min in 5x SSCT at RT on a nutator.

8. Hybridization

8.1 - Pre-hybridize worms in 180 µL of probe hybridization buffer for 30 min at 37°C in a thermal shaker, shaking at 700 rpm.

8.2 - Prepare probe solution by adding 1 pmol of each probe mixture (e.g. use 1 µL of 0.75 µM probe and 75 µL of probe hybridization buffer). Incubate at 37°C in a thermal shaker, shaking at 700 rpm.

8.3 - Remove the pre-hybridization solution and add the probe solution.

Biology Open • Supplementary information

8.4 - Incubate worms either for at least 2h or overnight (18-20 h) at 37°C in the thermal shaker, shaking at 700 rpm.

9. 1st wash

9.1 - Pre-heat probe wash buffer to RT. Then wash worms 4 x 30 min with 180 µL of probe wash buffer at RT, in the thermal shaker, shaking at 700 rpm.

9.2 - Wash the samples 2 x 5 min with 180 µL of 5×SSCT at RT, in the thermal shaker, shaking at 700 rpm.

Note: In case of nonspecificc, speckled signal in the epidermis, washing is not sufficient. The washes can be elongated further to eliminate nonspecifically bound probes.

10. Amplification

10.1 - To pre-amplify, remove 5x SSCT and add 180 µL of room temperature amplification buffer for at least 10 min at RT in the thermal shaker, shaking at 700 rpm.

10.2 - Prepare 6 pmol of hairpin h1 and 6 pmol of hairpin h2 by snap cooling 1.8 µL of the 3 µM hairpin stock: heat the samples at 95°C for 90 seconds and then cool them to room temperature in the dark for 30 min.

Note: Use PCR tubes/strips with 1 hairpin per tube.

10.3. - Prepare hairpin mixture by adding snap-cooled hairpins to 90 µL of amplification buffer.

10.4 - Add DAPI (1 ng/µL final concentration) to the hairpin mixture.

10.5 - Remove the pre-amplification solution and add the hairpin mixture. After this step, keep the samples in the dark.

10.6 - Incubate the worms overnight (16-20 h) in the dark at RT, in the thermal shaker, shaking at 700 rpm.

11. 2nd wash and post fixation

11.1 - Remove excess hairpins by washing with 180 µL of 5x SSCT in the thermal shaker at 700 rpm at RT:

- 2 x 5 min with 5x SSCT
- 1 x 30 min with 5x SSCT
- 1 x 5 min with 5x SSCT

11.2 - Postfix worms in 4% formaldehyde (in 5x-SSCT) for 10 min at RT, in the thermal shaker, shaking at 700 rpm

11.3 - Wash the worms with 0.125 M glycine in 5x SSCT for 10 min at RT, in the thermal shaker, shaking at 700 rpm.

11.4 - Wash the worms 3 x 5 min with 5x SSCT, in thermal shaker, shaking at 700 rpm.

11.5 - Wash the worms 3 x at least 30 min at RT, in the thermal shaker, shaking at 700 rpm.

12. Mounting

12.1 - Mount samples in ProLong Glass Antifade Mounting medium (Thermo Fisher Scientific, Cat No.: P36980) between two cover slips. Separate the cover slips using 0.1 mm spacers (SUNJin Lab, Cat. – No.: IS306).

13. Solutions & Material

13.1- General

- 10% SDS in ddH20, filtered
- PBSTx0.3 (0.3 % Triton-X100 in PBS):
    - 3 ml Triton-X100 in 1 L PBS
- 5 x SSCT 0.1x:
    - 50 ml 20 X SSC
    - 10 % Triton-X100 (2 ml)
    - Fill to 200 ml with ultrapure H2O
- 0.5 M citric acid (pH = 6):
    - 9.6 g citric acid in 75 ml H_2O
    - Bring to pH 6 (NaOH)
    - Fill to 100 ml, filter (0.2 µm filter membrane)
- 50 % dextran sulphate:
    - 50 g dextran sulphate powder
    - Fill to 80 ml with ultra pure water
    - Heat to 60 °C and stir or shake to dissolve
    - Fill to 100 ml with ultra pure water

Biology Open • Supplementary information

- Heparin (10 mg/ml, filtered)
- 50 x Denhardt's solution

13.2 - Fixation

- 5% NAC in PBS (prepare fresh):
    - 1 g NAC in 10 ml of PBS
    - 10 µl 10% Triton-X100 (filtered)
    - Fill to 20 ml with PBS
- Neutralized NAC (prepare fresh):
    - 8 ml H_2O
    - 1 ml of 5% NAC
    - 200 µl HEPES-NaOH (pH 7.25)
    - 5 µl phenol-red indicator solution
    - Adjust pH to ~7 with 1M NaOH (solution should turn light red; no full colour change)
    - Fill to 10 ml with H2O
- Fixation solution (4% paraformaldehyde in 0.5X PBSTx0.3, prepare fresh):
    - 10 ml 16% PFA
    - 20 ml PBSTx0.3
    - 10 ml H_2O
- 0.125 M glycine in PBSTx0.3
- 1x SSC

13.3 - Reduction

- Reduction solution (1 % NP-40, 0.5 % SDS, 50 mM DTT, 1x PBS):
    - 5 ml 10 % SDS
    - 1 ml NP-40
    - Fill to 95 ml with H_2O
    - Store at RT
    - Add 50 µL 1M DTT to 950 µL reduction solution directly before use

13.4 - Bleaching

Biology Open • Supplementary information

- Formamide bleaching solution (5% non-deionized formamide; 1.2% H2O2; in 0.5x SSC, prepare fresh):
    - 2.5 ml non-deionized formamide
    - 2 ml 30% H_2O_2
    - Fill to 50 ml with 0.5x SSC

13.5 - De- and rehydration

- 50% and 75% MeOH in PBSTx0.3

13.6 - Tissue permeabilization

- DEEP clear solution 1:
    - 4.5 ml of THEED (N, N, N', N'-Tetrakis(2-Hydroxyethyl)ethylene-diamin, Merk, Cat. No.: 87600)
    - 2.5 ml of Triton X-100
    - 12.5 g of urea filled to 50 ml with dH2O

13.7 - Hybridization

- Probe Hybridization Buffer
    - Formamide (30 %): 30 ml
    - 20x SSC (5x): 25 ml
    - 0.5 M citric acid (pH6, 9 mM): 1.8 ml
    - Tween20 (0.1%): 100 µL
    - 10 mg/ml heparin (50 µg/ml): 500 µL
    - 50x Denhardt's solution (1x) 2 ml
    - 50 % dextran sulphate in H_2O (5%): 10 ml
    - Fill to 100 ml with ultrapure H_2O
    - Aliquot in falcon tubes and store at -20 °C

13.8 - 1st wash

- Probe Wash Buffer
    - Formamide (30 %): 30 ml
    - 20x SSC (5x): 25 ml

Biology Open • Supplementary information

- 0.5 M citric acid (pH 6, 9 mM): 1.8 ml
- Tween20 (0.1%): 100 µL
- 10 mg/ml heparin (50 µg/ml): 500 µL
- Fill to 100 ml with ultrapure H_2O
- Aliquot in falcon tubes and store at -20 °C

13.9 – Amplification

- Amplification Buffer
  - 20x SSC (5x): 25 ml
  - Tween20 (0.1%): 100 µL
  - 50 % dextran sulphate in H_2O (5%): 10 ml
  - Fill to 100 ml with ultrapure H_2O
  - Aliquot in falcon tubes and store at -20 °C

13.10 - 2nd wash and post fixation

- 4% Formaldehyde solution: 10 ml 16 % PFA; fill to 40 ml with 5x SSCT
- 0.125 M glycine in 5x SSCT

13.11 - Mounting

- ProLong Glass Antifade Mountant (Thermo Fisher Scientific, Cat No.: P36980)

Biology Open • Supplementary information
