# Supplementary material for: Multiplexed gene expression analysis in dissociated and intact planarian flatworm tissues using in situ hybridization chain reaction
Source: Biol Open. 2026 Sep 10;15(9):bio062669. doi: 10.1242/bio.062669 (PMC13602376; doi:10.1242/bio.062669)
Supplement: Supplementary file 2 [file biolopen-15-062669_review_history.pdf]

Multiplexed gene expression analysis in dissociated and intact planarian flatworm tissues using in situ hybridization chain reaction

Fabienne Dreier, Peter Ditte, Philipp Weiss, Tobias Boothe and Jochen Christian Rink
10.1242/bio.062669

Editor: Tristan Rodríguez

Review timeline

Original submission:	29 April 2026
Editorial decision:	4 May 2026
First revision received:	22 July 2026
Accepted:	29 July 2026

Original submission

First decision letter

MS ID#: bio.062669

MS Title: Multiplexed gene expression analysis in dissociated and intact planarian flatworm tissues using in situ hybridization chain reaction

Authors: Fabienne Dreier, Peter Ditte, Philipp Weiss, Tobias Boothe and Jochen Christian Rink

I have now reached a decision on the above manuscript.

The reviewer reports are shown at the bottom of this email.

As you will see, the reviewers gave favourable reports, but raised some critical points that will require amendments to your manuscript. I hope that you will be able to carry these out, because we would like to be able to accept your paper.

At this stage, we also ask you to ensure your manuscript complies with our formatting guidelines - please see our manuscript preparation guidelines for details. Provided you are able to fully address the referees' comments, we are positive about publication of your paper (we accept over 95% of revision submissions) and therefore hope you won't mind any extra work involved in reformatting your manuscript at this point.

Please upload both a 'clean' version of your Word file, along with a highlighted version clearly showing where you have made changes in the revised manuscript. Please avoid using 'Track changes' in Word files as these are lost in PDF conversion.

I should be grateful if you would also provide a point-by-point response detailing how you have dealt with the points raised by the reviewers in the 'Response to Reviewers' box. Please attend to all of the reviewers' comments. If you do not agree with any of their criticisms or suggestions please explain clearly why this is so.

Reviewer 1

Comments for the author

© 2026. Published by The Company of Biologists under the terms of the Creative Commons Attribution License (https://creativecommons.org/licenses/by/4.0/).

This is a well-executed and clearly presented manuscript describing the optimisation of an HCR protocol for planarians. The study is timely and well justified, as current FISH-based approaches remain limited in multiplexing capacity and can be labour-intensive. The authors take a systematic and logical approach, beginning with dissociated cells and progressing to whole-mount animals, including regeneration paradigms (e.g. anteriorised/posteriorised animals following RNAi). The validation of marker expression against previously published patterns is convincing and supports the robustness of the method. Overall, this represents a valuable technical advance that will be of clear interest to the planarian community.

I have a few points that would strengthen the manuscript further:

1. "Data not shown" statements
There are several instances throughout the manuscript where conclusions are supported by "data not shown" (e.g. wash optimisation, DEEP Clear S2 usage). Given that this is a methods-focused study, these optimisation steps are central to the value of the work. I strongly recommend that these data be included, at least in the Supplementary Information, to improve transparency and reproducibility.

2. Quantification and statistical analysis
The manuscript would benefit from clearer reporting of sample sizes (e.g. number of animals, preparations, or cells analysed) for each experiment. In addition, details of the statistical analysis should be provided, including whether comparisons were formally tested, and whether differences are statistically significant.

In summary, this is a strong and useful methodological contribution. Addressing the points above would further enhance the impact and reproducibility of the work.

<u>Reviewer 2</u>

Comments for the author

The paper by Jochen Rink and colleagues describes a newly established protocol in the lab to use in intact and dissociated planaria (a well-known model system to address the cellular, molecular and genetic mechanisms underlying whole-body regeneration) HCR (in situ hybridization chain reaction) to simultaneously analyse expression of multiple genes in control condition or in functional genetic experiments. As a proof of principle, authors first demonstrate that HCR can be used in dissociated planaria cells (Figure 1). Then, they optimize the HCR protocol (originally described for mouse samples) by the use of the DEPP Clear S1 (instead of detergent or proteinase K), as well some changes in the protocol to improve signal/noise ratio and reduce protocol length (eg. temperature changes, shorter probe exposure and longer washes, Figure 2). They then compare different clearing and mounting media and demonstrate that the refined protocol can be used to identify changes in gene expression as a consequence of genetic manipulation. The paper is well written and figures are self-explanatory. Since this is a "methods" paper, my only three comments for improvement would be the following ones:
(1) to include a Table where the whole protocol (or at least, the most important aspects) are clearly depicted and
(2) to better describe in the Methods sections all the protocols and avoid using cryptic descriptions such as "done as described in ….·"
(3) to include the classical references by the Baguña and Salo labs that demonstrated that neoblasts can be depleted by irradiation and that planaria is a good model system to address regeneration.

Reviewer's Responses to Questions

Experimental quality
Are experiments performed using appropriate methods that will answer the question (or test the hypothesis or support the observations) posed by the authors? Is the right tool used for the job?
If 'No', please indicate reasons in Comments for Author box below.
Reviewer #1:
- Yes

© 2026. Published by The Company of Biologists under the terms of the Creative Commons Attribution License (https://creativecommons.org/licenses/by/4.0/).

Reviewer #2:

- Yes

Were the data analyzed using appropriate statistical tests?
If 'No', please indicate reasons in Comments for Author box below.
Reviewer #1:

- No

Reviewer #2:

- Yes

Reproducibility
Were experiments performed using adequate number of biological replicates?
If 'No', please indicate reasons in Comments for Author box below.
Reviewer #1:

- Yes

Reviewer #2:

- Yes

Is there sufficient raw data to assess the rigor of the analysis?
If 'No', please indicate reasons in Comments for Author box below.
Reviewer #1:

- Yes

Reviewer #2:

- Yes

Does the methods section provide sufficient detail to permit reproducibility?
If 'No', please indicate reasons in Comments for Author box below.
Reviewer #1:

- Yes

Reviewer #2:

- No

Completeness
Are the manuscript's conclusions supported by the data?
If 'No', please indicate reasons in Comments for Author box below.
Reviewer #1:

- Yes

Reviewer #2:

- Yes

Scholarship
Do the authors cite and discuss the merits of data that would argue for and against their conclusion?
If 'No', please indicate reasons in Comments for Author box below.
Reviewer #1:

- Yes

© 2026. Published by The Company of Biologists under the terms of the Creative Commons Attribution License (https://creativecommons.org/licenses/by/4.0/).

Reviewer #2:

- Yes

Does the manuscript title & abstract accurately reflect the contents of the manuscript, without hyperbole?
If 'No', please indicate reasons in Comments for Author box below.
Reviewer #1:

- Yes

Reviewer #2:

- Yes

First revision

Author response to reviewers' comments

We thank the reviewers for their time and very constructive feedback. As detailed below, we have added an additional figure and table and made numerous edits to the text, figure legends, and references to address reviewer suggestions (all changes are highlighted in yellow). We believe our revised manuscript is considerably improved as a result.

Reviewer 1

This is a well-executed and clearly presented manuscript describing the optimisation of an HCR protocol for planarians. The study is timely and well justified, as current FISH-based approaches remain limited in multiplexing capacity and can be labour-intensive. The authors take a systematic and logical approach, beginning with dissociated cells and progressing to whole-mount animals, including regeneration paradigms (e.g. anteriorised/posteriorised animals following RNAi). The validation of marker expression against previously published patterns is convincing and supports the robustness of the method. Overall, this represents a valuable technical advance that will be of clear interest to the planarian community.

Thank you for your positive assessment of our work.

I have a few points that would strengthen the manuscript further:

1. "Data not shown" statements
There are several instances throughout the manuscript where conclusions are supported by "data not shown" (e.g. wash optimisation, DEEP Clear S2 usage). Given that this is a methods-focused study, these optimisation steps are central to the value of the work. I strongly recommend that these data be included, at least in the Supplementary Information, to improve transparency and reproducibility.

- We replaced all the "data not shown" statements with references to figures or supplementary figures in our revised manuscript. We also added an additional supplementary figure illustrating the effects of proteinase K and Deep Clear S2 on signal intensity (Figure S3) and expanded the graph in Figure 2B to include the missing comparisons.

2. Quantification and statistical analysis
The manuscript would benefit from clearer reporting of sample sizes (e.g. number of animals, preparations, or cells analysed) for each experiment. In addition, details of the statistical analysis should be provided, including whether comparisons were formally tested, and whether differences are statistically significant.

© 2026. Published by The Company of Biologists under the terms of the Creative Commons Attribution License (https://creativecommons.org/licenses/by/4.0/).

- Done. Our revised manuscript now includes the exact sample sizes for every condition in the figure legends. For statistical analyses, we added t-tests to compare two conditions and ANOVAS to compare multiple conditions, with asterisks added to the graphs in Figure 2, Figure 4, Figure S3, and Figure S4 to denote statistical significance.

In summary, this is a strong and useful methodological contribution. Addressing the points above would further enhance the impact and reproducibility of the work.

Reviewer 2

The paper by Jochen Rink and colleagues describes a newly established protocol in the lab to use in intact and dissociated planaria (a well-known model system to address the cellular, molecular and genetic mechanisms underlying whole-body regeneration) HCR (in situ hybridization chain reaction) to simultaneously analyse expression of multiple genes in control condition or in functional genetic experiments. As a proof of principle, authors first demonstrate that HCR can be used in dissociated planaria cells (Figure 1). Then, they optimize the HCR protocol (originally described for mouse samples) by the use of the DEPP Clear S1 (instead of detergent or proteinase K), as well some changes in the protocol to improve signal/noise ratio and reduce protocol length (e.g. temperature changes, shorter probe exposure and longer washes, Figure 2). They then compare different clearing and mounting media and demonstrate that the refined protocol can be used to identify changes in gene expression as a consequence of genetic manipulation. The paper is well written and figures are self-explanatory.

Thank you!

Since this is a "methods" paper, my only three comments for improvement would be the following ones:
(1) to include a Table where the whole protocol (or at least, the most important aspects) are clearly depicted

Thank you for this suggestion and done. We added a table (Table 1), as suggested (see below), to provide an overview of the major steps of the protocol and the time required for each. Additionally, we added a “Detailed bench protocols” section with the detailed protocol as it is used in our laboratory.

Table 1: Protocol timeline

Day	Procedure	Time required
1	1.1 Fixation	0.5 h
	1.2 Bleaching	1.5 h - 2 h
	1.3 Dehydration	Over night
2	2.1 Rehydration	0.5 h
	2.2 Tissue permeabilization (DEEP Clear S1)	0.5 h
	2.3 Hybridization	2 h
	2.4 Washing	2.5 h
	2.5 Amplification	Over night
3	3.1 Washing + post fixation	3 h
	3.2 Mounting (Pro Long Antifade Glass)	2h curation

(2) to better describe in the Methods sections all the protocols and avoid using cryptic descriptions such as "done as described in …"

- Done. We rewrote the relevant parts of the method section to include detailed descriptions of the cited protocols. Readers no longer have to consult other papers to look up parts of the methods.

(3) to include the classical references by the Baguña and Salo labs that demonstrated that neoblasts can be depleted by irradiation and that planaria is a good model system to address regeneration.

© 2026. Published by The Company of Biologists under the terms of the Creative Commons Attribution License (https://creativecommons.org/licenses/by/4.0/).

- We added references to Saló & Baguñà, 2002 and to Baguñà, Saló, and Auladell, 1989.

Second decision letter

MS ID#: bio.062669R1

MS Title: Multiplexed gene expression analysis in dissociated and intact planarian flatworm tissues using in situ hybridization chain reaction

Authors: Fabienne Dreier, Peter Ditte, Philipp Weiss, Tobias Boothe and Jochen Christian Rink

I am happy to tell you that your manuscript has been accepted for publication in Biology Open, pending our standard publication integrity checks. It was accepted on 29th July 2026.

© 2026. Published by The Company of Biologists under the terms of the Creative Commons Attribution License (https://creativecommons.org/licenses/by/4.0/).
